# COVID 19 Vaccine for Adolescents. Concern about Myocarditis and Pericarditis

**DOI:** 10.3390/pediatric13030061

**Published:** 2021-09-01

**Authors:** Giuseppe Calcaterra, Jawahar Lal Mehta, Cesare de Gregorio, Gianfranco Butera, Paola Neroni, Vassilios Fanos, Pier Paolo Bassareo

**Affiliations:** 1 Department of Cardiology, Postgraduate Medical School of Cardiology, University of Palermo, 90127 Palermo, Italy; peppinocal7@gmail.com; 2 Department of Medicine, University of Arkansas for Medical Sciences and the Veterans Affairs Medical Center, Little Rock, AR 72205, USA; mehtajl@uams.edu; 3 Department of Clinical and Experimental Medicine, University of Messina, 98125 Messina, Italy; cesare.degregorio@unime.it; 4 Cardiology, Cardiac Surgery, and Heart Lung Transplantation Department, ERN, GUAR HEART, Bambino Gesu’ Hospital and Research Institute, IRCCS Rome, 00165 Rome, Italy; gianfranco.butera@opbg.net; 5 Neonatal Intensive Care Unit, Department of Surgical Sciences, Policlinico Universitario di Monserrato, University of Cagliari, 09042 Monserrato, Italy; paolaneroni123@gmail.com (P.N.); vafanos@tiscali.it (V.F.); 6 Department of Cardiology, Mater Misericordiae University Hospital and Our Lady’s Children’s Hospital Crumlin, University College of Dublin, School of Medicine, D07R2WY Dublin, Ireland

**Keywords:** vaccine, myocarditis, pericarditis, multi-system inflammatory disease, Kawasaki-like syndrome, SARS-CoV-2

## Abstract

The alarming onset of some cases of myocarditis and pericarditis following the administration of Pfizer–BioNTech and Moderna COVID-19 mRNA-based vaccines in adolescent males has recently been highlighted. All occurred after the second dose of the vaccine. Fortunately, none of patients were critically ill and each was discharged home. Owing to the possible link between these cases and vaccine administration, the US and European health regulators decided to continue to investigate the potential causal relationship between COVID-19 mRNA vaccines and myocarditis. In any case, none of the patients fulfilled the criteria for multi-system inflammatory syndrome or Kawasaki-like disease and there was no evidence of acute SARS-CoV-2 infection.

## 1. Introduction

Infection caused by the severe acute respiratory syndrome coronavirus 2 (SARS-CoV-2, COVID-19) has spread across the globe, causing almost 4 million deaths. Most reports relating to COVID-19 have focused primarily on adults, whereas several questions about children and adolescents infected by SARS-CoV-2 still remain unanswered [1,2].

By 13 May 2021, coronavirus infected 3.94 million children and sent more than 16,000 to hospital; these numbers exceed those of people usually hospitalized for flu in an average year, according to the American Academy of Pediatrics [3]. About 316 children died of COVID-19 in the United States, making it one of the top 10 causes of death in children since the pandemic began. At the time of this report, it appears that severe illness due to COVID-19 is rare among children. However, more and more data on the long-term impact of the pandemic are needed, including those about the long-term physical health of infected children, as well as the “emotional and mental health” effects [2,3,4,5].

## 2. Vaccines: Myocarditis and Pericarditis

All vaccines against SARS-CoV-2 were initially authorized, with unprecedented speed, for emergency use only in adult people over 18 years of age. As a commendable result, all vaccines undoubtedly saved many thousands of lives [6]. On 10 May 2021, the US Food and Drug Administration (FDA) authorized the Pfizer–BioNTech vaccine for emergency use in children aged 12 and older. This vaccine proved its favourable safety profile and efficacy against COVID-19, in some multinational, placebo-controlled, observer-blinded randomised trials [7]. On 28 May 2021, the European Union medicines regulator agency (EMA) authorized the Pfizer–BioNTech COVID-19 vaccine for use in children aged 12 to 15 [8].

More than 161 million people in the United States have received at least one dose of a COVID-19 mRNA vaccine and about 4.5 million of them were between 12 and 18 years of age.

On 22 May 2021, the Centers for Disease Control and Prevention (CDC) in the USA reported the onset of some cases of myocarditis and pericarditis in young adults after the administration of the COVID-19 mRNA-based vaccine by Pfizer–BioNTech [7]. No similar cases were reported after administration of the Janssen COVID-19 vaccine (Johnson & Johnson), according to the agency’s vaccine safety group [9].

In early July, the EMA’s Pharmacovigilance Risk Assessment Committee (PRAC) said that they were investigating reports of myocarditis/pericarditis after receiving the Pfizer–BioNtech vaccine, but did not report any concerns, since such incidents were occurring at similar rate in the general population [10]. On 3 June 2021, Israeli health regulators found 275 cases of myocarditis between December 2020 and May 2021 among more than 5 million vaccinated people who had received the Pfizer COVID-19 vaccine. All the cases were likely linked to the previous vaccination [11,12].

On 10 June 2021, the CDC vaccine adverse events reporting system (VAERS) released a detailed sentence, as follows: “The cases seem to have occurred predominantly in young adults (475 suspected myocarditis involving people younger than 30 years; 79 cases reported were in patients 16 or 17 years old and 196 cases among young adults aged 18–24). This incidence of myocarditis is higher than expected for this age group, about four days after their second dose of BNT162b2 mRNA (Pfizer-BioNTech) and the mRNA-1273 (Moderna) vaccines. The disease process was more common in males than in females. Cases of pericarditis occurred as well within several days after mRNA COVID-19 vaccination” [13,14,15,16].

The CDC also stated that these “relatively few” cases of myocarditis which were reported to VAERS after receiving a COVID-19 vaccine, should be determined to discover whether they meet the diagnostic criteria for myocarditis. Any direct connection to a COVID-19 vaccination should be proved, because myocarditis and pericarditis are also typical inflammatory features in response to an infection or to some other triggers.

In any case, the CDC posted guidance on its website urging physicians to be alert to unusual heart symptoms, which could include chest pain, shortness of breath, or palpitations, among young people who have just received their shots. Nonetheless, most cases appear to be mild, and their follow up showed no evidence of acute SARS-CoV-2 infection and did not fulfil the diagnostic criteria for MIS-C [7,13,17].

Despite these reports, the CDC strongly recommend vaccines against SARS-CoV-2 for children aged 12 and older, emphasizing the rarity of myocarditis. The severity of cases of myocarditis and pericarditis following the vaccination can vary. Most cases responded well to the usual medical treatment and rest. In confirmed cases, elevated serum troponin, an altered ECG and an abnormal cardiac MRI in terms of patchy delayed late gadolinium enhancement consistent with myocarditis and myocardial oedema were found [7]. Of note, acute infection by coronavirus itself can cause myocarditis, including the persistent syndrome called “long COVID” [1,5,18,19]. In this young population, coronary events are less likely to be a source of these symptoms. It is important to rule out other potential causes of myocarditis and pericarditis, such as infections with entero-virus and respiratory-virus [20].

The true incidence of myocarditis is unknown, and varies on the basis of season, geography and age. It has been reported to occur in approximately 1.95/100,000 children <15 years of age in Finland and in 2.16 cases per 100,000 US military service members. It is more common in males and generally reveals a bimodal incidence pattern, with peaks at <2 years of age and again in adolescence [16,19,21,22].

Myocarditis has been reported as an adverse event following other vaccinations, such as the smallpox vaccine, which is a live vaccine. Thus, if myocarditis following SARS-CoV-2 mRNA vaccination was confirmed to be causally related, it would be unique among non-live vaccines. Understanding of the underlying mechanism and possible late sequelae is crucial.

Parents should still vaccinate their children, because the benefits of vaccination in this population against this highly transmissible disease exceed the risks of rare transient adverse events. Other known risks of COVID-19, including Kawasaki-like/MIS-C, are much greater [1,21,22,23,24]. However, with schools soon to reopen worldwide, and children being possible carriers of SARS-CoV-2, much clarification on this issue is needed as soon as possible [25].

## 3. Conclusions

Certain characteristics are pointing toward a “rare, but real” clinical entity of myocarditis/pericarditis following COVID-19 vaccination. First, the events occur within days since vaccination. Second, they tend to be more common in males and in younger people. Third, the number of adverse events is greater than the so-called “standard myocarditis incidence rate”. We feel that there should be at least a year of study and follow up from vaccination in clinical trials, the amount of data typically essential for full approval, instead of the 2 months required for emergency use authorization [26].

At the moment, three questions remain open and unanswered, namely: why is myocardium a side effect? Why are adolescent males affected the most? Why is the onset after the second dose of m-RNA vaccine?

## Data Availability

Not applicable.
